# Pelvic lymph node decidua mimicking metastatic cervical cancer in pregnancy

**DOI:** 10.1136/ijgc-2022-003544

**Published:** 2022-05-31

**Authors:** Krista S Pfaendler, H James Williams

**Affiliations:** 1 Gynecologic Oncology, West Virginia University Cancer Institute, Morgantown, West Virginia, USA; 2 Obstetrics and Gynecology, West Virginia University School of Medicine, Morgantown, West Virginia, USA; 3 Pathology, Anatomy, and Laboratory Medicine, West Virginia University School of Medicine, Morgantown, West Virginia, USA

**Keywords:** Cervical Cancer, Pathology, Lymph Nodes, Pregnancy Complications, Neoplastic

Cervical cancer is the most common gynecologic malignancy during pregnancy with a 10-year incidence of only 3.3 per 100 000 births in the USA.^1^ In patients with squamous cell carcinoma of the cervix during pregnancy, decidual cell reaction has been described in pelvic lymph nodes^2^ and para-aortic lymph nodes^3^ at the time of radical hysterectomy. In patients with International Federation of Gynecology and Obstetrics (FIGO) 2018 stage IA2–IB2 tumors, radical hysterectomy and lymphadenectomy may be performed immediately following cesarean delivery once fetal maturation is confirmed.^4^

In this case, we demonstrate decidualized tissue within pelvic lymph nodes of a patient with squamous cell carcinoma of the cervix who underwent a classical cesarean section followed by modified radical hysterectomy with bilateral pelvic lymphadenectomy at 36 weeks and 3 days gestational age. Her cervical tumor measured 2.3 cm with middle third cervical stromal invasion and lymphovascular invasion present. Macroscopically, the lymph nodes were normal in appearance. However, microscopically, the pelvic lymph nodes had prominent nests of cells with abundant eosinophilic cytoplasm in a subcapsular and sinusoidal pattern concerning for metastatic squamous cell carcinoma (Figure 1A, B). These cells are contrasted with the primary cervical squamous cell carcinoma (Figure 2A, B). Immunohistochemical staining showed the cells within the lymph nodes to be negative for pancytokeratin (AE1/AE3; Figure 3A), p40, CK5/6, CK8/18, p63 (Figure 3B), p16, and S-100. The cells within the lymph nodes were positive for estrogen and progesterone receptors (Figure 3C) with focal CD10 positivity (Figure 3D). Concurrent studies performed on the cervical squamous cell carcinoma confirmed positivity for p63, CK5/6 and p16.

**Figure 1:**
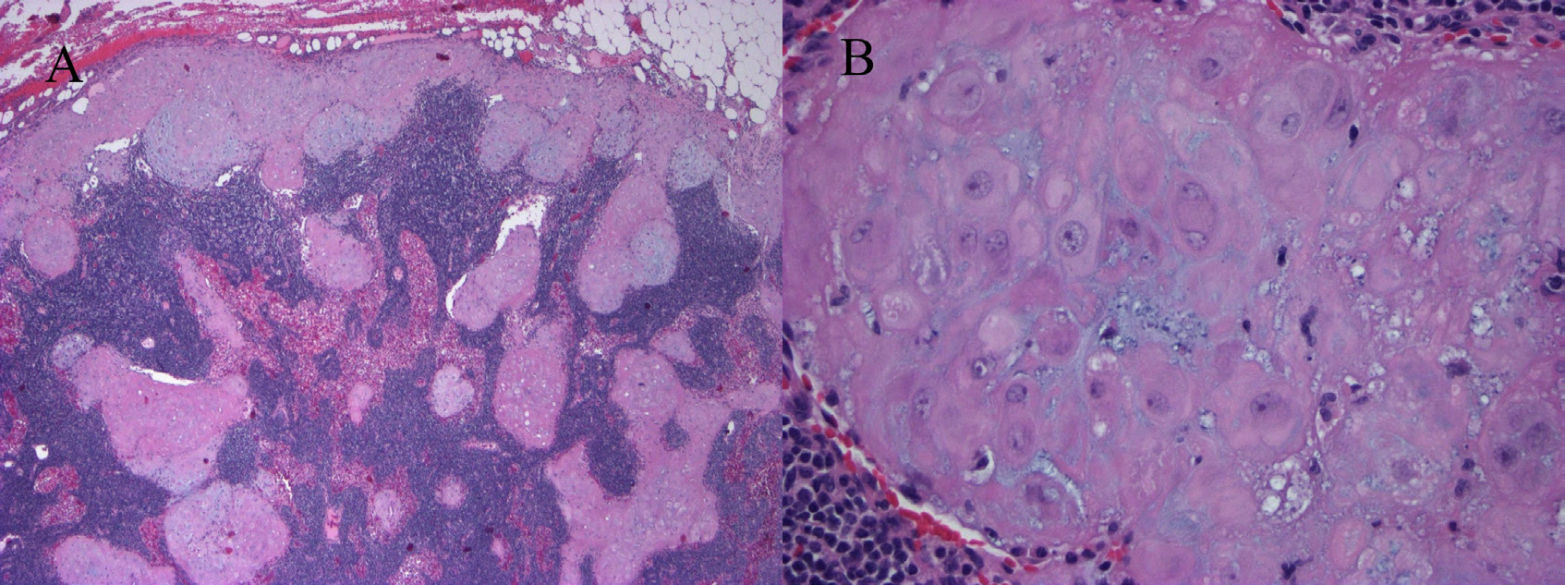
Pelvic lymph node. (A) Prominent nests of cells with abundant eosinophilic cytoplasm in a subcapsular and sinusoidal pattern (H&E, x4). (B) Enlarged vesicular nuclei with prominent nucleoli and abundant eosinophilic cytoplasm; mitoses absent (H&E, x40).

**Figure 2:**
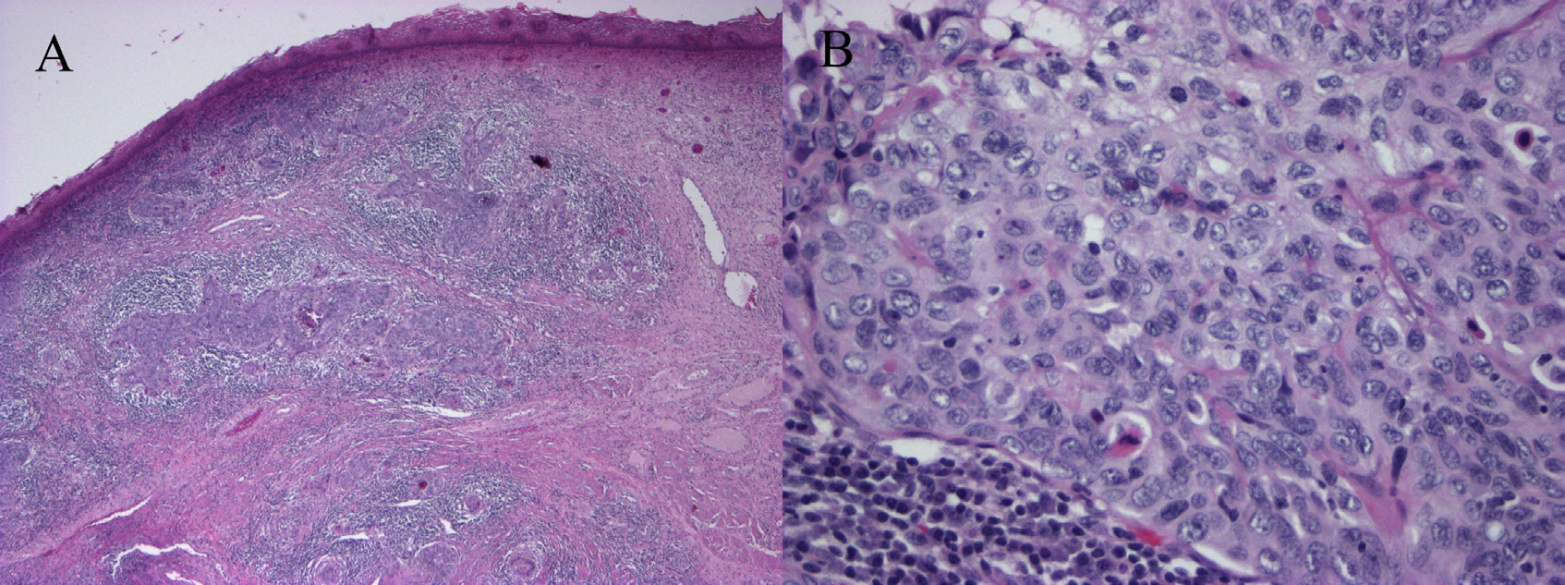
Cervix. (A) Invasive moderately differentiated squamous cell carcinoma (H&E, x4). (B) Enlarged vesicular nuclei with prominent nucleoli and moderate eosinophilic cytoplasm; mitoses present (H&E, x40).

**Figure 3:**
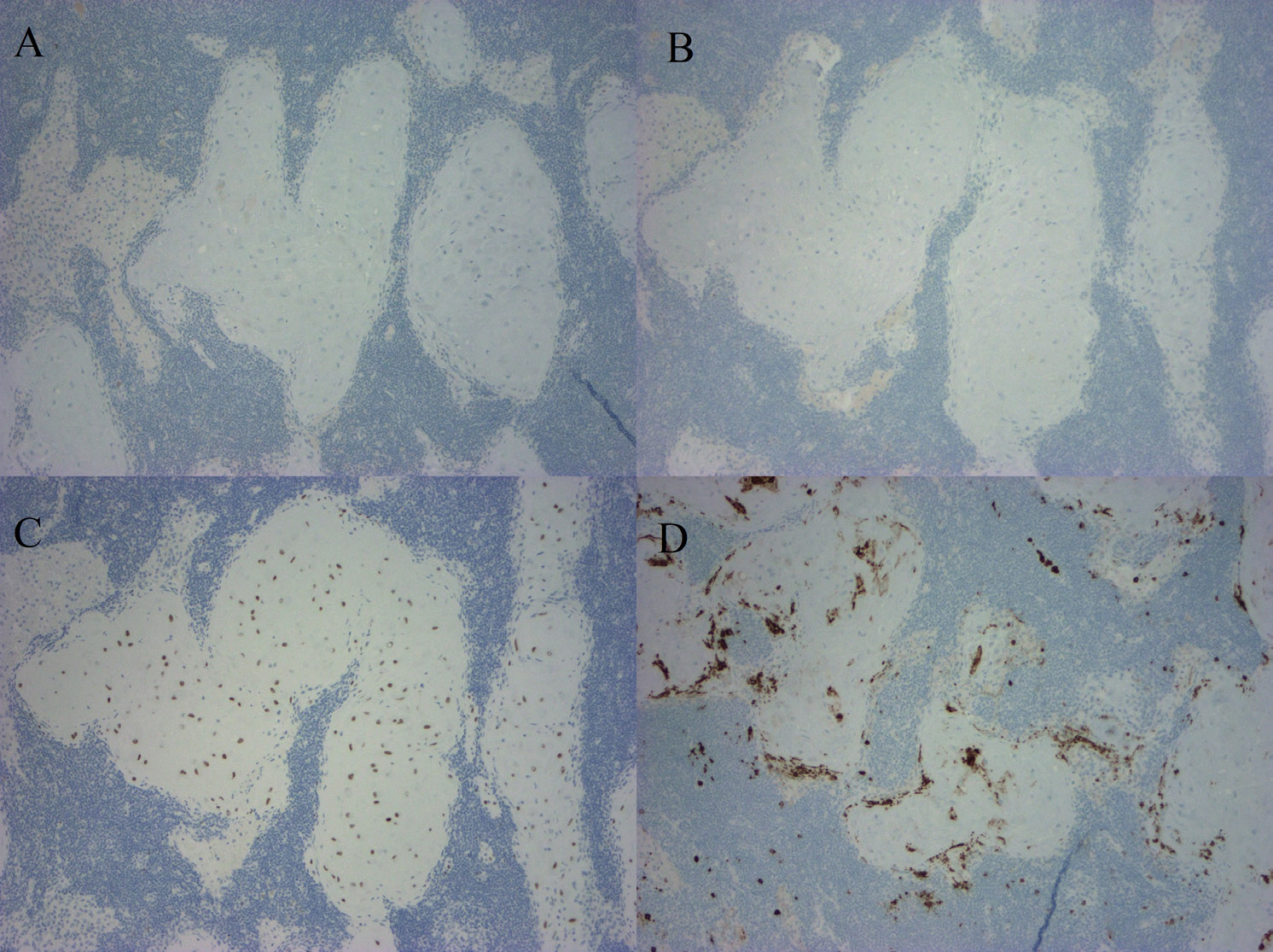
Pelvic lymph node. (A) Negative for pancytokeratin (AE1/AE3). (B) Negative for p63. (C) Positive for progesterone receptor. (D) Positive for CD10 (immunostains, x10).

The histopathological and immunohistochemical findings were consistent with decidua mimicking metastatic squamous carcinoma in the pelvic lymph nodes. Etiologic considerations of decidua in lymph nodes include decidualization of pre-existing endometriosis and lymphatic embolization. While ectopic decidua does not have clinical significance, if this finding is not recognized and nodes are incorrectly interpreted as containing metastatic disease, patients may receive adjuvant treatment not otherwise indicated.

## Data Availability

All data relevant to the study are included in the article or uploaded as supplementary information.
